# A comprehensive map of preferentially located motifs reveals distinct proximal *cis*-regulatory sequences in plants

**DOI:** 10.3389/fpls.2022.976371

**Published:** 2022-10-12

**Authors:** Julien Rozière, Cécile Guichard, Véronique Brunaud, Marie-Laure Martin, Sylvie Coursol

**Affiliations:** ^1^ Université Paris-Saclay, CNRS, INRAE, Université Evry, Institute of Plant Sciences Paris-Saclay (IPS2), Gif sur Yvette, France; ^2^ Université de Paris Cité, Institute of Plant Sciences Paris-Saclay (IPS2), Gif sur Yvette, France; ^3^ Université Paris-Saclay, INRAE, AgroParisTech, Institut Jean-Pierre Bourgin (IJPB), Versailles, France; ^4^ Université Paris-Saclay, INRAE, AgroParisTech, UMR MIA-Paris-Saclay, Palaiseau, France

**Keywords:** gene expression, *cis*-regulatory elements, preferentially located motifs, gene-proximal regions, gene regulatory network, plant

## Abstract

Identification of *cis*-regulatory sequences controlling gene expression is an arduous challenge that is being actively explored to discover key genetic factors responsible for traits of agronomic interest. Here, we used a genome-wide *de novo* approach to investigate preferentially located motifs (PLMs) in the proximal *cis*-regulatory landscape of *Arabidopsis thaliana* and *Zea mays*. We report three groups of PLMs in both the 5’- and 3’-gene-proximal regions and emphasize conserved PLMs in both species, particularly in the 3’-gene-proximal region. Comparison with resources from transcription factor and microRNA binding sites shows that 79% of the identified PLMs are unassigned, although some are supported by MNase-defined cistrome occupancy analysis. Enrichment analyses further reveal that unassigned PLMs provide functional predictions that differ from those derived from transcription factor and microRNA binding sites. Our study provides a comprehensive map of PLMs and demonstrates their potential utility for future characterization of orphan genes in plants.

## Introduction

As sessile organisms, plants, must adapt to local constraints such as bacteria, fungi, and pests, as well as to environmental changes. One of the fundamental drivers of their adaptation is the activation or repression of gene transcription (Waters et al., 2017; Alonge et al., 2020; Azodi et al., 2020; Liu et al., 2020; Zhou et al., 2022). These processes are tuned by numerous *cis*-regulatory DNA sequences, the characterization of which is a central question for the complete understanding of transcriptional response mechanisms [for a recent review, see (Schmitz et al., 2021)]. Numerous experimental and predictive efforts (Lai et al., 2019; Savadel et al., 2021; Schmitz et al., 2021) have been made to characterize them, highlighting several *cis*-regulatory regions. In addition to the distal *cis*-regulatory DNA sequences, which include enhancers (Fagny et al., 2021) and can be more than 1 Mbp away from their target gene, there are the 5’- and 3’-gene-proximal regions that are rich in *cis*-regulatory DNA sequences (Li et al., 2012; Wallace et al., 2014; Zemlyanskaya et al., 2021).

The 5’-gene-proximal region is located in the bases framing the transcription start site (TSS) and includes the core promoter and the promoter. The core promoter is directly involved in the binding of the transcription initiation complex and is by definition essential for gene expression. The promoter is a region upstream of the core promoter that is involved in binding of many additional transcription factors (TFs) that can modulate basal gene expression (Schmitz et al., 2021). The 3’-gene-proximal region, also called terminator, is a *cis*-regulatory region that is strongly involved in regulating gene expression, but unlike the 5’-gene-proximal region, it has been little studied (Mayr, 2019; Bernardes and Menossi, 2020). It frames the transcription termination site (TTS) and is known to influence gene transcription termination by allowing the binding of the cleavage and polyadenylation complex (CPMC). This region is also rich in TF binding sites (TFBSs) and may interact with the 5’-gene-proximal region through the phenomenon of gene looping (Wang et al., 2010). Despite our current knowledge of these regions fundamental to gene function, our understanding remains incomplete, and much effort is still required to achieve their complete characterization at the genome level.

Interestingly, the *cis*-regulatory DNA sequences in these two gene-related regions appear to be associated with fixed topological constraints. This observation holds for all core promoter sequences with motifs such as the TATA-box, which is located about 30 bases upstream of the TSS (Yamamoto et al., 2007; Bernard et al., 2010; Jores et al., 2021). This phenomenon is also observed in the promoters of several plant species for TFBSs, which occupy preferential position depending on the associated TF family (Yu et al., 2016; Ksouri et al., 2021). Finally, the sites involved in CPMC binding in the 3’-gene-proximal region also show topological constraints with respect to the TTS (Bernardes and Menossi, 2020). Based on this biological context and in order to contribute to a better characterization of these gene-proximal regions in plants, we propose to use and extend an *in silico* method called PLMdetect (Preferentially Located Motif detection) (Bernard et al., 2010). Originally, this method aimed to identify DNA motifs in *Arabidopsis thaliana* that are overrepresented at a specific position compared with TSS and are therefore referred to as preferentially located motifs (PLMs) (Bernard et al., 2010; Bueso et al., 2014; Frei dit Frey et al., 2014; Martínez et al., 2015).

Here, we performed a genome-wide and *de novo* PLMdetect-based study of the 5’ and 3’-proximal regions of genes from *A. thaliana* and *Zea mays*. We aimed to determine the extent of which their differences in genome content and genome architecture were reflected in the characteristics of their PLMs in both gene-proximal regions. Our results revealed the organizing principle of the plant PLM landscape and provide a valuable resource for the characterization of unannotated genes in plants.

## Results

### Implementation of large-scale and *de novo* PLM detection

**Figure 1:**
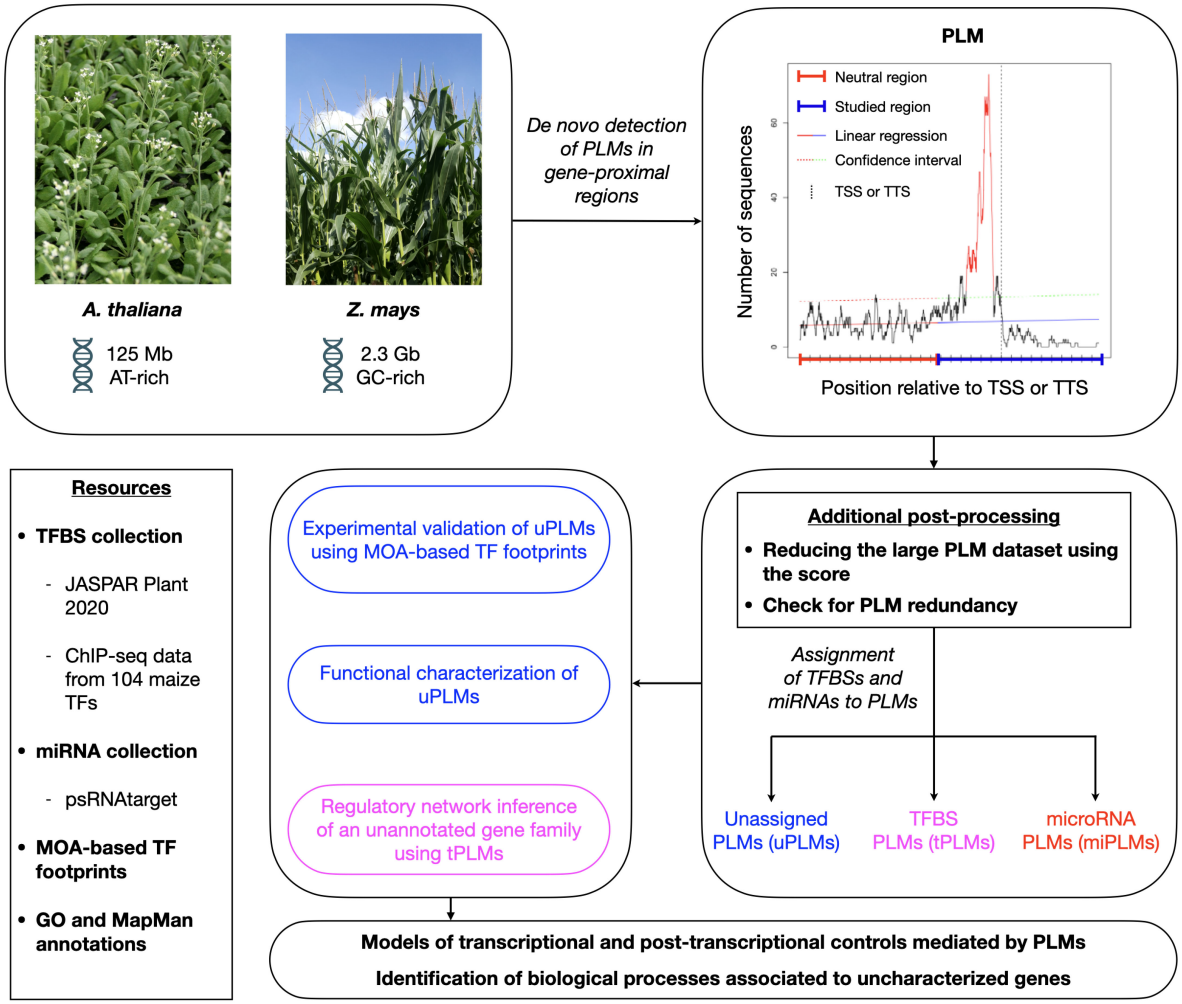
Schematic overview of the workflow used in this study for de novo and genome-wide quantification, characterization, and exploitation of PLMs in gene-proximal regions of *A. thaliana* and *Z. mays*. We first detected PLMs in the 5’- and 3’**-**proximal regions of genes from both plant species. Next, we selected the PLMs according to their score, checked for PLM redundancy and determined whether some of the detected PLMs might be TFBSs (referred to as tPLMs) or targeted by miRNAs (referred to as miPLMs) using distinct resources of TFBSs [JASPAR Plant 2020 (Fornes et al., 2019) and top 1% k-mers from ChIP-seq data of 104 maize TFs (Tu et al., 2020)] and miRNA binding sites [psRNATarget (Dai et al., 2018)]. Unassigned PLMs (referred to as uPLMs)-containing gene sets were functionally characterized with GO and MapMan enrichments relative to the genome. Moreover, we showed that some 5’-uPLMs were supported by MOA-based TF footprints data (Savadel et al., 2021) of Z. mays. Using 5’-tPLMs, we finally inferred the regulatory network of a poorly characterized maize-specific gene family. Collectively, our results provide functional inside into the regulatory role of PLMs and highlight biological processes associated to uncharacterized genes.

To define the PLM profile associated with the 5’- and 3’-gene-proximal regions, we extended the PLMdetect method (Bernard et al., 2010). Given the 5’-gene-proximal regions and a motif, this method first calculates the number of motif occurrences at each position in the sequence to obtain a motif distribution. Second, a linear regression is calculated for a neutral region defined as the first 500 bp of the 5’-gene-proximal region where no accumulation of PLMs is expected. Third, in the region under study, the predicted values are calculated with a confidence interval of 99%. If the observed occurrence distribution exceeds the confidence interval, the motif is considered as a PLM. Thus, a PLM is visually defined by a motif distribution that has a peak in the region under study, indicating that it is statistically overrepresented at a preferential distance from the TSS. The PLM is characterized by (i) its preferential position, defined as the position of the peak’s top, (ii) a functional window, defined as the portion located between the peak boundaries, and (iii) a score defined as the difference between the peak’s top value and the upper bound of the confidence interval at the preferential position. To implement large-scale and *de novo* PLM detection, we also investigated the 3’-gene-proximal regions by computing the motif distribution according to the TTS and considered all non-polymorphic DNA 4-mers to 8-mers (**Figure 1**).

### Genome-wide PLM identification in gene-proximal regions of *A. thaliana* and *Z. mays*

**Figure 2:**
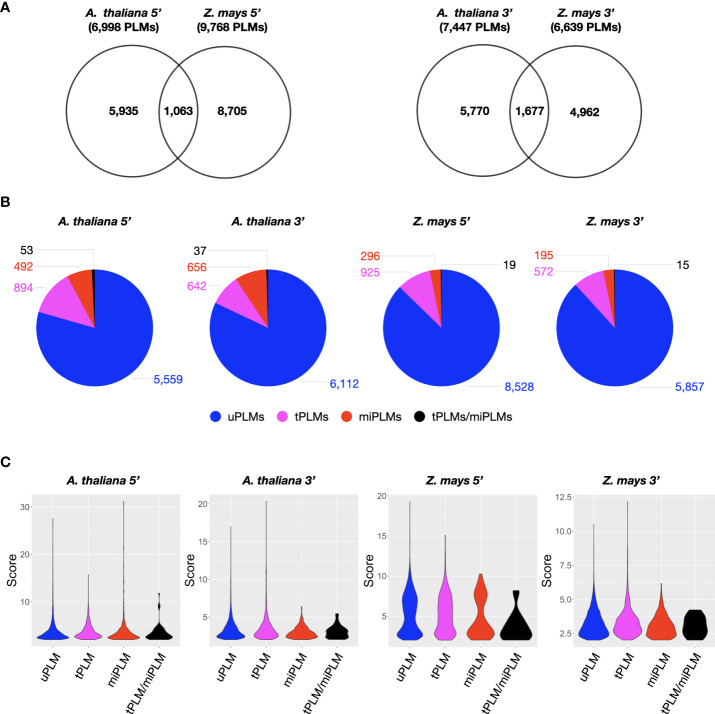
Characterization of PLM content in gene-proximal regions of *A. thaliana* and *Z. mays.* **(A)** Venn diagram showing the extent of overlap between 5’- or 3’-PLMs of *A. thaliana* and *Z. mays*. **(B)** Dissection of PLM types identified in the 5’- or 3’-gene-proximal region of *A. thaliana* and Z. mays. **(C)** Violin plot of PLM scores according to the PLM types in the 5’- or 3’-gene-proximal region of *A. thaliana* and *Z. mays*.

Distribution by score values revealed two populations of PLMs with a score less than or greater than 2 in each gene-proximal region of each species (**Supplementary Figure 1**). A score greater than 2 indicates a position where the occurrence of the motif is very high compared with the value calculated from the neutral region. In addition, the PLM subpopulation described by a score above 2 was smaller than the one with a score below 2. Both arguments led us to consider only the PLM population with a score above 2 to characterize the 5’- and 3’-gene-proximal regions. We identified 6,998 and 9,768 (7,447 and 6,639) PLMs in the 5’ (3’)-gene-proximal regions (referred to as 5’ (3’)-PLMs) of *A. thaliana* and *Z. mays*, respectively (**Figure 2A** and **Supplementary Table 1**). To verify that detected PLMs were not redundant, we tested the inclusion relationship between two PLMs (a k-mer included in a larger k-mer) if they shared 50% of their functional windows and occurred in almost the same gene sets (Jaccard index >= 0.9) (**Supplementary Table 2**). Only 84 PLM pairs corresponding to 159 5’-PLMs of *Z. mays* had the same PLM-containing gene sets. This meant that a maximum of 79 PLMs could be filtered, i.e. 0.8% of the 5’-PLMs detected in *Z. mays*. Therefore, we considered the redundancy of PLMs to be negligible and retained our original number of PLMs based on the score for all subsequent analyses.

**Figure 3:**
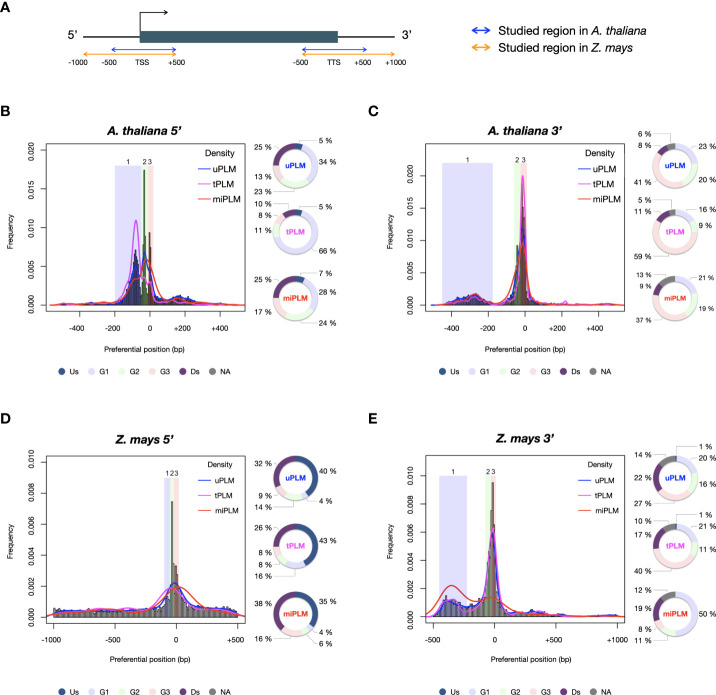
PLM frequency and characterization according to their preferential position. **(A)** Schema of the studied regions with respect to the gene in *A. thaliana* and Z. mays. **(B)** 5’-PLMs in *A. thaliana.* Group 1: [-200;-50[; Group 2: [-50;-10[; Group 3: [-10;+20[. **(C)** 3’-PLMs in *A. thaliana.* Group 1]:-450;-175]; Group 2]:-60;-25]; Group 3]:-25;+10]. **(D)** 5’-PLMs in *Z. mays.* Group 1: [-100;-50[; Group 2: [-50;-20[; Group 3: [-20;+20[. **(E)** 3’-PLMs in *Z. mays.* Group 1]:-450;-225]; Group 2]:-75;-30]; Group 3]:-30;+10 bp]. Us: region located upstream the three groups; G1, G2 and G3: groups 1, 2 and 3; Ds: region located downstream the three groups; NA: region located between the groups when they are not juxtaposed.

Comparison of the PLM content of the two species revealed that *A. thaliana* and *Z. mays* shared 1,063 5’-PLMs and 1,677 3’-PLMs (**Figure 2A**). It is worth noting that 98% of these PLMs were located in the 200 bases around the TSS or the TTS. Examination of the preferred position of the PLMs also revealed three visually distinguishable groups within each target region of each species with similar distribution patterns (**Figure 3**). In the 5’-gene-proximal region, groups 1 (*A. thaliana*: ]-450;- 175]; *Z. mays*: ]-450;-225]) and 2 (*A. thaliana*: ]-60;-25]; *Z. mays*: ]-75;-30]) were localized upstream of the TTS, while group 3 (*A. thaliana*: ]-25;+10]; *Z. mays*: ]-30;+10 bp]) was localized on the TTS. We also found that 72% of 5’-PLMs in *Z. mays* were localized upstream and downstream of the identified groups, whereas in *A. thaliana* 72% of the 5’-PLMs were localized in groups.

Additionally, each group of 5’-PLMs had specific nucleotide content. Group 1 was composed of A, T, C and G nucleotides in equal proportions in both species. In contrast, group 2 was composed predominantly of A/T (74% and 64% in *A. thaliana* and *Z. mays*, respectively) in agreement with previous observations reporting TATA and TATA-like boxes in this region (Joshi, 1987). In the case of group 3, we found that the GC content of the 5’-PLMs differed between the two species (37% of GC in *A. thaliana vs* 55% in *Z. mays*), in agreement with the report of GC-rich genes in monocot species (Clément et al., 2014; Sundararajan et al., 2016) and recent promoter comparisons using *A. thaliana* and the three cereal species brachypodium, wheat and barley (Peng et al., 2016).

For the 3’-PLMs of both species, we found that groups 2 and 3 consisted predominantly of A/T nucleotides (>70%), whereas group 1 in both species consisted mainly of C/G nucleotides (>60%). We did also observe that 3,877 and 2,130 3’-PLMs detected in the [-40;+10] bp interval relative to the TTS (which corresponds to the end of group 2 and the whole group 3) in *A. thaliana* and *Z. mays*, respectively, showed similarities to the *cis*-elements that guide the CPMC essential for mRNA biogenesis (Bernardes and Menossi, 2020). These 3’-PLMs were A/T-rich (over 78%) consistently with the far upstream element (FUE) and near upstream element (NUE). Furthermore, those localized 10 bases upstream and downstream of the TTS were composed of sequences rich in T (42% in both species) >A (38% in *A. thaliana* and 34% in *Z. mays*) >C (11% in *A. thaliana* and 14% in *Z. mays*) >G (9% in *A. thaliana* and 11% in *Z. mays*), in agreement with the known proportions of nucleotides in the cleavage element (CE) (Bernardes and Menossi, 2020).

### Identification and positional distribution of TFBS-like PLMs in gene-proximal regions

We anticipated that some of the detected PLMs might be TFBSs. Although the genomes of *A. thaliana* and *Z. mays* are distant, the TFBSs of orthologous TFs are found to be similar in sequence (Tu et al., 2020). Therefore, we decided to use a common resource of plant TFBSs from experimental data to assign our PLMs. This consisted of the JASPAR Plant 2020 database (Fornes et al., 2019) in combination with the top 1% k-mers from ChIP-seq data of 104 maize TFs (Tu et al., 2020). It led to the discovery that 13.5% of the 5’-PLMs (9.1% of the 3’-PLMs) of *A. thaliana* were indeed similar in sequence to known TFBS and were therefore referred to as tPLMs (**Figure 1A**, **Figure 2B** and **Supplementary Tables 3A, B**). In *Z. mays*, 9.6% of the 5’-PLMs and 8.8% of the 3’-PLMs corresponded to tPLMs (**Figure 2B** and **Supplementary Tables 3C, D**). To evaluate these tPLM predictions, we used the experimental *A. thaliana* ChIP-seq and DAP-seq data integrated into the ReMap database (Hammal et al., 2022). We found that 61% of 5’-tPLMs and 55% of 3’-tPLMs of *A. thaliana* were covered by experimental peaks for the corresponding TFs, supporting our TFBS assignment of PLMs (**Supplementary Tables 3A, B**). It is worth noting that in *A. thaliana* 66% of the 5’-tPLMs were localized in group 1, whereas 59% of the 3’-tPLMs were localized in group 3 (**Figures 3B, C**). In Z. mays, 26% of the 5’-tPLMs were localized upstream and 35% downstream of the identified groups, whereas the 3’-tPLMs followed the same behavior as in *A. thaliana*, with greater localization in group 3. Overall, these results show strong localization of the 5’-tPLMs in the interval between 200 and 50 bp upstream of TSS in agreement with previous observations in *A. thaliana* and *Prunus Persica* (Yu et al., 2016; Ksouri et al., 2021). In contrast, the 3’-tPLMs mainly localized in the TTS region in both species.

**Figure 4:**
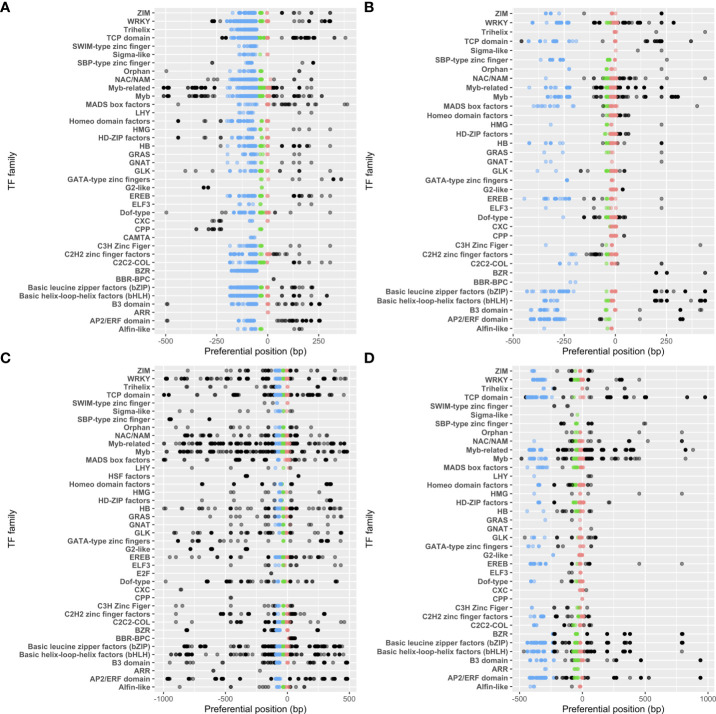
tPLM preferential positions per TF family. tPLMs in 5’- **(A)** and 3’- **(B)** gene-proximal regions of *A. thaliana*. tPLMs in 5’- **(C)** and 3’- **(D)** gene proximal regions of *Z. mays*. Blue, green and red points correspond to the tPLMs in groups 1, 2 and 3, respectively. The black points correspond the tPLMs that do not belong to any group. The opacity of the points is relative to the number of tPLMs at that position.

We next investigated how the different TF families were distributed in each proximal region. Among the 47 TF families listed in our reference, 39 and 40 (35 and 37) were susceptible to bind to 5’ (3’)-tPLMs in *A. thaliana* and *Z. mays*, respectively (**Figure 4**). We observed that all TF families associated to 3’-PLMs also targeted 5’-PLMs. We also noted that some TF families were detected only with the 5’- or 3’-tPLMs of *A. thaliana* or *Z. mays* (**Figure 4A**). Using the ReMap data, we determined whether the lack of detection of these TF families was also observed in experimental data from *A. thaliana*. In contrast to predictions, we found that these families indeed bind experimentally to these regions, indicating that their TFBSs have fewer topological constraints. Additionally, we found that all TF families were not similarly distributed in each gene-proximal region. For example, the MYB TFs had tPLMs in all three groups of each region and species studied (**Figure 4**). In contrast, the Trihelix TFs was only present in group 1 in the 5’-proximal region of *A. thaliana*. Other TF families, such as the G2-like TFs, were likely to target different number of PLM groups according to the region and species considered.

### PLMs occur at microRNA binding sites in gene-proximal regions

Previous studies showed that microRNAs (miRNAs) can target transcripts with sequence complementarity (Bartel, 2009), thus inducing their degradation. It was also described in *Brassica* that miRNA methylates the promoter region of *SP11* gene to silence it (Tarutani et al., 2010). Hence, we predicted that 5’- and 3’-PLMs could be associated to miRNA binding sites. Using the plant small RNA target analysis server psRNATarget (Dai et al., 2018)”. we found that 7.8% and 3.2% (9.3% and 3.2%) of the 5’ (3’)-PLMs can be targeted by miRNAs (referred to as 5’ (3’)-miPLMs) in *A. thaliana* and *Z. mays*, respectively (**Figure 1**, **Figure 2B** and **Supplementary Table 4**). To assess the quality of the predictions, we performed full-length miRNA alignment at miPLM sites, allowing 0-5 mismatches as described previously for miRNA-target interactions (Ossowski et al., 2008; Axtell, 2013). We found that 51% and 29% of 5’-miPLMs (55% and 41% of 3’-miPLMs) in *A. thaliana* and *Z. mays*, respectively, corresponded to full-length miRNAs (**Supplementary Tables 4**). We further noticed that 5’-miPLMs had a maximum density downstream of the TSS, which is consistent with the main mode of action of miRNAs, and supports our approach and findings (**Figures 3B, D**). Surprisingly, more than half of the 5’-miPLMs of *A. thaliana* were located in groups 1 and 2 (**Figure 3B**), while those of *Z. mays* were overwhelmingly found outside the groups (**Figure 3D**). We also noticed that 3’-miPLMs were more localized in group 3 than in the other two groups in *A. thaliana* (**Figure 3C**), while half of them were found in group 1 in *Z. mays* (**Figure 3E**).

**Figure 5:**
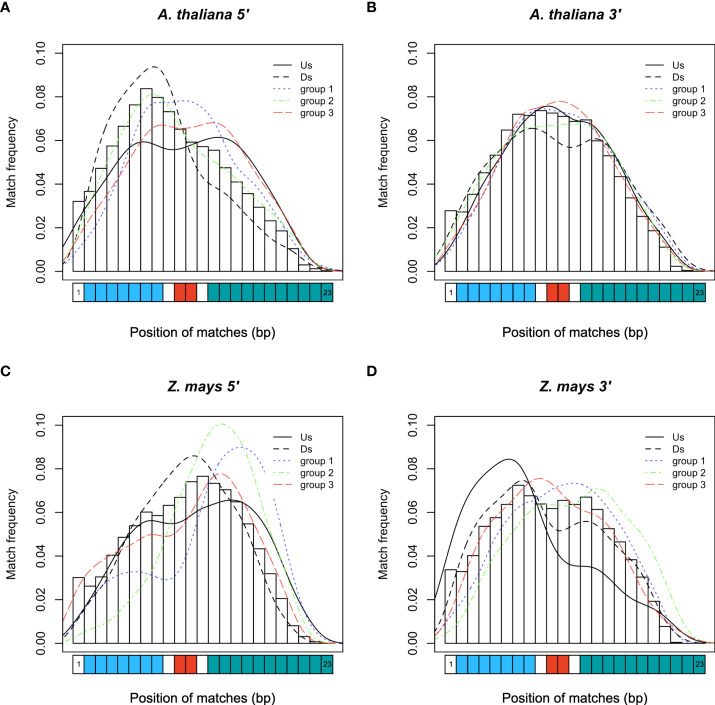
Frequency of miRNA bases covered by miPLMs. **(A)** Frequencies for the 5’-miPLMs and the **(B)** 3’-miPLM of *A. thaliana*. **(C)** Frequencies for the 5’-miPLMs and the **(D)** 3’-miPLM of *Z. mays*. The color curves indicate the densities of matched-ribonucleotide positions depending on whether the miPLM that matches belongs to one of the PLM groups (groups 1, 2 or 3) or is located upstream (Us) or downstream (Ds) of these groups. The blue, red and green rectangles on the abscissa represent the bases of the 5’- seed region, the cleavage site and the 3’-compensatory end of the miRNA, respectively.

We then investigated which sequence of miPLMs was homologous to that of miRNAs, since the latter are composed of different parts that do not all have the same function (Ossowski et al., 2008). It is known that the 5’-seed region (positions 2-8) of miRNAs is involved in target recognition, and the cleavage site (positions 10-11) is also critical for post-transcriptional regulation. Therefore, we characterized the coverage of PLMs-miRNA homologies (**Figure 5**). We found that 5’-miPLMs from *A. thaliana* had more frequent homologies with the 5’-seed region, whereas those from *Z. mays* had more frequent homologies with the compensatory 3’-end (**Figure 5**). For 3’-miPLMs, homologies were more frequent in the center of the miRNA, surrounding the cleavage site in both species, e frequent homologies in the 5’ seed region, whereas those belonging to any of the three groups had higher homology frequencies in the bases surrounding the cleavage site in both species, suggesting that miPLMs have distinct functions depending on the region and species considered.

Interestingly, we found that 53 and 19 5’-miPLMs (37 and 15 3’-miPLMs) corresponded to tPLMs in *A. thaliana* and *Z. may*s, respectively (**Figure 2B** and **Supplementary Tables 1A-D**). In *A. thaliana*, we noticed that WRKY, Basic helix-loop-helix factors (bHLH) and Basic leucine zipper factors (bZIP) represented the three major TF families identified in the 5’-gene-proximal region, while C2H2 zinc finger factors, Myb-related and HD-ZIP factors were the three major TF families identified in the 3’-gene-proximal region (**Supplementary Tables 4E, F**). In *Z. mays*, bHLH, bZIP and BZR represented the three major TF families identified in the 5’-gene-proximal region, while bHLH, bZIP and TCP domain were the major TF families in the 3’-gene-proximal region (**Supplementary Tables 4G, H**).

### Unassigned PLMs are putative *cis*-regulatory players

Comparison with resources of TF and miRNA binding sites revealed that more than 79% of the identified PLMs were unassigned PLMs (referred to as uPLMs) (**Figures 1**, **2B, C** and **Supplementary Table 1**). To determine whether uPLMs with the strongest topological constraints (score > 10) were only core promoter motifs (i.e., motifs bound by the transcription machinery), we evaluated the score of uPLMs and the content of RNA polymerase II binding site. We found that 39.2% and 83.1% of the major 5’-uPLMs detected in *A. thaliana* and *Z. mays*, respectively, were distinct from RNA polymerase II binding sites (11.5% and 6.9% of the 5’-uPLMs detected in *A. thaliana* and *Z. mays*, respectively) (Fornes et al., 2019), suggesting that uPLMs may contain *cis*-regulatory players (**Figure 2C** and **Supplementary Table 5**).

In *A. thaliana*, one-third of the 5’-uPLMs were localized in group 1, while more than one-third of the 3’-uPLMs were localized in group 3. In *Z. mays*, the 5’-uPLMs were preferentially localized upstream and downstream of the three groups detected, showing a greater dispersion than that observed in *A. thaliana*. Furthermore, the 3’-uPLMs of *Z. mays* had a more balanced distribution among the different groups and downstream part than those of *A. thaliana*. Interestingly, the density of the 5’-uPLMs in both species was higher in the core promoter region corresponding to group 2 and known to be the locus of many regulatory events (Grosschedl and Birnstiel, 1980; Molina and Grotewold, 2005; Yamamoto et al., 2007; Bernard et al., 2010), confirming the relevance of our hypothesis (**Figure 3**).

Recently, MNase-defined cistrome-occupancy analysis (MOA-seq) to identify chromatin-accessible regions in developing maize ears led to the identification of 215 small (<30 bp) TF footprints distributed in total across 100,000 non-overlapping binding sites in the genome (Savadel et al., 2021). Given the relatively small size of these footprints and their remarkable clustering within 100 bp proximal to the promoters, we examined them for sequence and position (**Figure 1**). We found that 85 of these 215 TF footprints significantly matched 203 of our motifs. Considering the position of these motifs (plus or minus 30 bases upstream and downstream of the corresponding PLM functional window), 30% of them covered 79 PLMs (**Supplementary Table 9**), including 19 tPLMs, 13 miPLMs and 50 uPLMs. Overall, these results support our hypothesis that uPLMs comprise putative *cis*-regulatory players.

### uPLMs provide specific functional predictions

**Figure 6:**
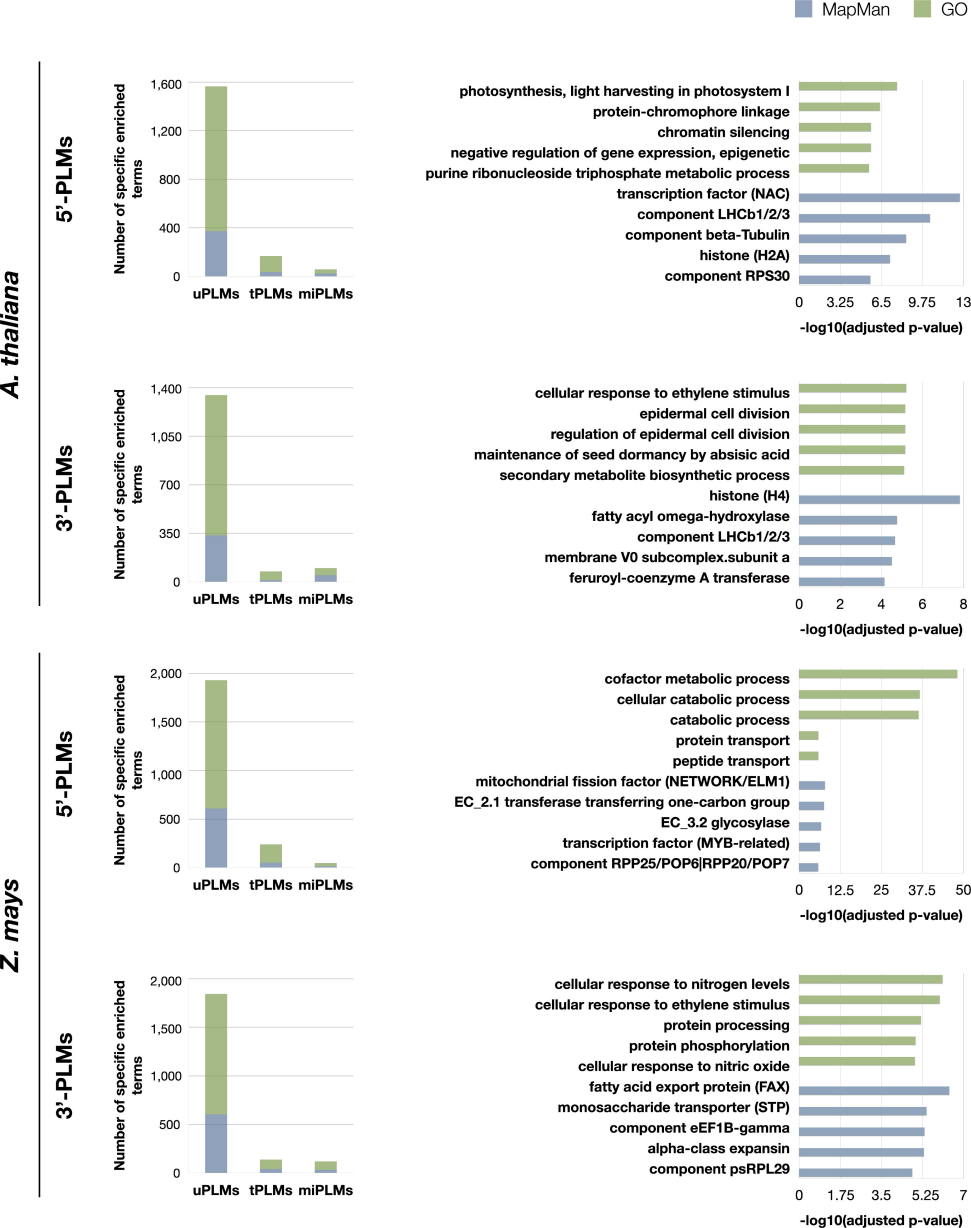
GO and MapMan terms enriched specifically for each type of PLMs-containing gene sets. Values for GO and Mapman terms are shown in green and blue, respectively. On the left: histograms of the number of GO and MapMan terms enriched specifically for each type of PLMs-containing gene sets in the two species studied. On the right: the 5 most enriched terms specifically for uPLMs. The bar values of the histograms indicate the -log(adjusted p-values) for each term. Map-Man terms have been truncated at the maximum precision level. The corresponding integer MapMan terms are as follows: transcription factor (NAC): RNA biosynthesis. transcriptional regulation.transcription factor (NAC); component LHCb1/2/3: Photosynthesis.photophosphorylation.photosystem II.LHC-II complex.component LHCb1/2/3; component beta-Tubulin: Cytoskeleton organisation.microtubular network.alpha-beta-Tubulin heterodimer.component beta-Tubulin; histone (H2A): Chromatin organization. histones.histone (H2A); component RPS30: Protein biosynthesis.ribosome biogenesis.small ribosomal subunit (SSU).SSU proteome.component RPS30; histone (H4): Chromatin organisation.histones.histone (H4); fatty acyl omega-hydroxylase: Cell wall organisation.cutin and suberin.cuticular lipid formation.fatty acyl omega-hydroxylase; membrane V0 subcomplex.subunit a: Solute transport.primary active transport.V-type ATPase complex.membrane V0 subcomplex.subunit a; feruroyl-coenzyme A transferase: Cell wall organisation.cutin and suberin.alkyl-hydrocinnamate biosynthesis.feruroyl-coenzyme A transferase; mitochondrial fission factor (NETWORK/ELM1): Cell cycle organisation.organelle division.mitochondrion and peroxisome division.mitochondrial fission factor (NETWORK/ELM1); EC_2.1 transferase transferring one-carbon group: Enzyme classification.EC_2 transferases.EC_2.1 transferase transferring one-carbon group; EC_3.2 glycosylase: Enzyme classification.EC_3 hydrolases.EC_3.2 glycosylase; transcription factor (MYB-related): RNA biosynthesis.transcriptional regulation.MYB transcription factor superfamily.transcription factor (MYB-related); component RPP25/POP6|RPP20/POP7: RNA processing.ribonuclease activities.RNA-dependent RNase P complex.component RPP25/POP6|RPP20/POP7; fatty acid export protein (FAX): Lipid metabolism.lipid trafficking.fatty acid export protein (FAX); monosaccharide transporter (STP): Solute transport.carrier-mediated transport.MFS superfamily. SP family.monosaccharide transporter (STP); component eEF1B-gamma: Protein biosynthesis.translation elongation.eEF1 aminoacyl-tRNA binding factor activity.eEF1B eEF1A-GDP-recycling complex.component eEF1B-gamma; alpha-class expansin: Cell wall organisation.cell wall proteins.expansin activities.alpha-class expansin; component psRPL29 Protein biosynthesis.organelle machinery.plastidial ribosome.large ribosomal subunit proteome.component psRPL29.

To characterize further the uPLMs, we used GO-term and MapMan functional category enrichment analysis to classify them according to the genes in which they occur (**Figure 1**). In both species, the 5’- and 3’-uPLMs-containing gene sets constituted two highly differentiated populations in terms of their biological processes or MapMan categories relative to the other identified PLM classes, further confirming that they include *cis*-regulatory players (**Figure 6** and **Supplementary Tables 6, 7**).

Comparing 5’- and 3’-uPLMs-containing gene sets revealed specific terms associated with each of the two sets (**Supplementary Figure 2** and **Table 7**). Notably, we observed that “cellular response to ethylene stimulus” was one of the five most enriched GO terms in the 3’-uPLMs-containing gene set of both *A. thaliana* and *Z. mays*. Some of the genes considered are characterized by uPLMs signals in the -450 to -200 bases relative to the TTS. These signals are further supported by the fact that the uPLM sequences are very conserved between the two species (CGTCG and its reverse-complementary CGACG for *A. thaliana*; ACGCCCAC/GGGCGTCC and its reverse-complementary GGACGCCC for *Z. mays*). Terms related to “Cell wall organisation” were also present in the five most enriched MapMan terms for the *A. thaliana*-uPLMs-containing gene set in both regions, although the protein classes identified were different. For example, we found that part of the genes encoding alpha-expansin are characterized by 5’-uPLMs signals localized after the TSS, while part of the genes encoding acyl omega-hydroxylases are characterized by 3’-uPLMs signals localized in group 1 and 2.

As expected, each species had also specific enriched terms (**Supplementary Figure 2 and Table 7**). For the 5’-uPLMs-gene set, these terms were mainly related to “cell killing” in *A. thaliana*, while they were associated with “transposition” and antibiotic metabolic/catabolic processes in *Z. mays* (**Supplementary Tables 8G, H**). For the 3’-uPLMs-gene set, specific enriched terms were once again related to “cell killing” in *A. thaliana*, while they were mainly related to “translation” in Z. mays. Taken together, these findings reveal that uPLMs provide functional predictions that differ from those derived from tPLMs and miPLMs (**Figure 6** and **Supplementary Tables 6-8**).

### Biological processes associated to uncharacterized genes through integration of PLM information

**Figure 7:**
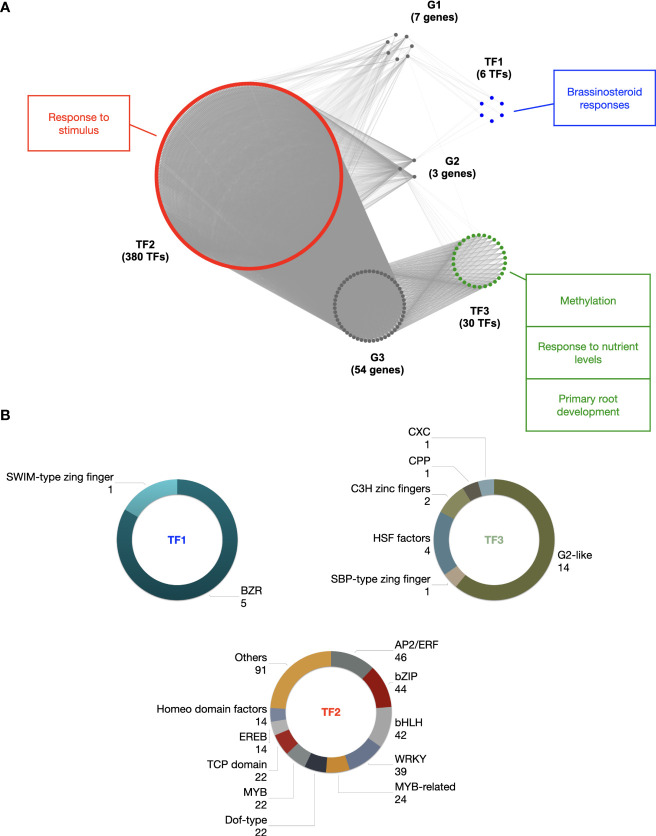
Gene regulatory network of the HOM04M002476 gene family of *Z. mays.* **(A)** Topological structure of the gene regulatory network of the HOM04M002476 gene family. G1, G2 and G3 are the three HOM04M002476 gene modules (grey) identified by the LBM procedure. TF1 (red), TF2 (green) and TF3 (blue) are the three TF modules identified by the LBM procedure potentially involved in the regulation of HOM04M002476 genes. GO enrichments of TF modules are indicated with corresponding colors. **(B)** TF families associated to the three TF modules.

Taking into account the contribution of PLMs, we thought to use them to infer gene regulatory networks and go further and deeper into the characterization of some gene families (**Figure 1**). We focused on a poorly characterized, *Z. mays*-specific gene family (referred to as HOM04M002476 by PLAZA) defined only by the GO term “transposition” (**Supplementary Table 8G**). This gene family consists of 65 genes, 64 of which were considered in the detection of 5’-PLMs (**Supplementary Table 11A**). Using the 5’-tPLMs detected for all these 64 genes, we investigated the TF-target gene relationships (**Figure 7A**). A total of 545 tPLMs were associated with 416 TFs belonging to 37 distinct TF families. Among them, AP2/ERF domain, Myb-related and WRKY were the three most abundant TF families (**Supplementary Table 11**).

Clustering based on latent block model (LBM) revealed three modules of target genes referred to as G1, G2 and G3 with 7, 3 and 54 members, respectively (**Figure 7A** and **Supplementary Table 11**). It also revealed three modules of TFs referred to as TF1, TF2 and TF3 with 6, 380 and 30 TFs belonging to 2, 29 and 6 TF families, respectively (**Figures 7A, B** and **Supplementary Table 11**). We found that genes belonging to module G1 were regulated by TFs from modules TF1 (2/2 families), TF2 (16/29 families) and TF3 (1/6 family). It is worth noting that the SWIM-type zinc finger TF family of module TF1 was specific to genes from G1 module. Genes belonging to module G2 were also regulated by TFs belonging to all three modules, including the BZR TF family of module TF1, all TF families of module TF2, and the HSF factors and G2-like TF families of module TF3. In contrast, genes belonging to module G3 were only regulated by TFs from modules TF2 and TF3. Furthermore, the CXC, CPP and SBP-type zinc finger TF families of module TF3 covered specifically genes from G3 module.

To elucidate the potential involvement of this gene regulatory network in biological processes, we conducted GO and MapMan enrichment analysis of the TF modules. As expected, terms enriched in a common way in all three modules were related to the regulation of transcription (**Supplementary Table 12**). Additionally, in module TF1, the most specifically enriched terms were related to brassinosteroid responses (**Figure 7A** and **Supplementary Tables 12A, B**). In module TF2, except for terms related to transcriptional regulation, the most enriched terms was related to “response to stimulus” (**Figure 7A**; **Supplementary Tables 12C, D**). Finally, in module TF3 the most enriched terms were mainly related to methylation, response to nutrient levels and primary root development (**Figure 7A** and **Supplementary Tables 12E, F**). Our computational approach therefore paves the way for pinpointing the function of this gene family in these distinct processes in future follow-up studies.

## Discussion

**Figure 8:**
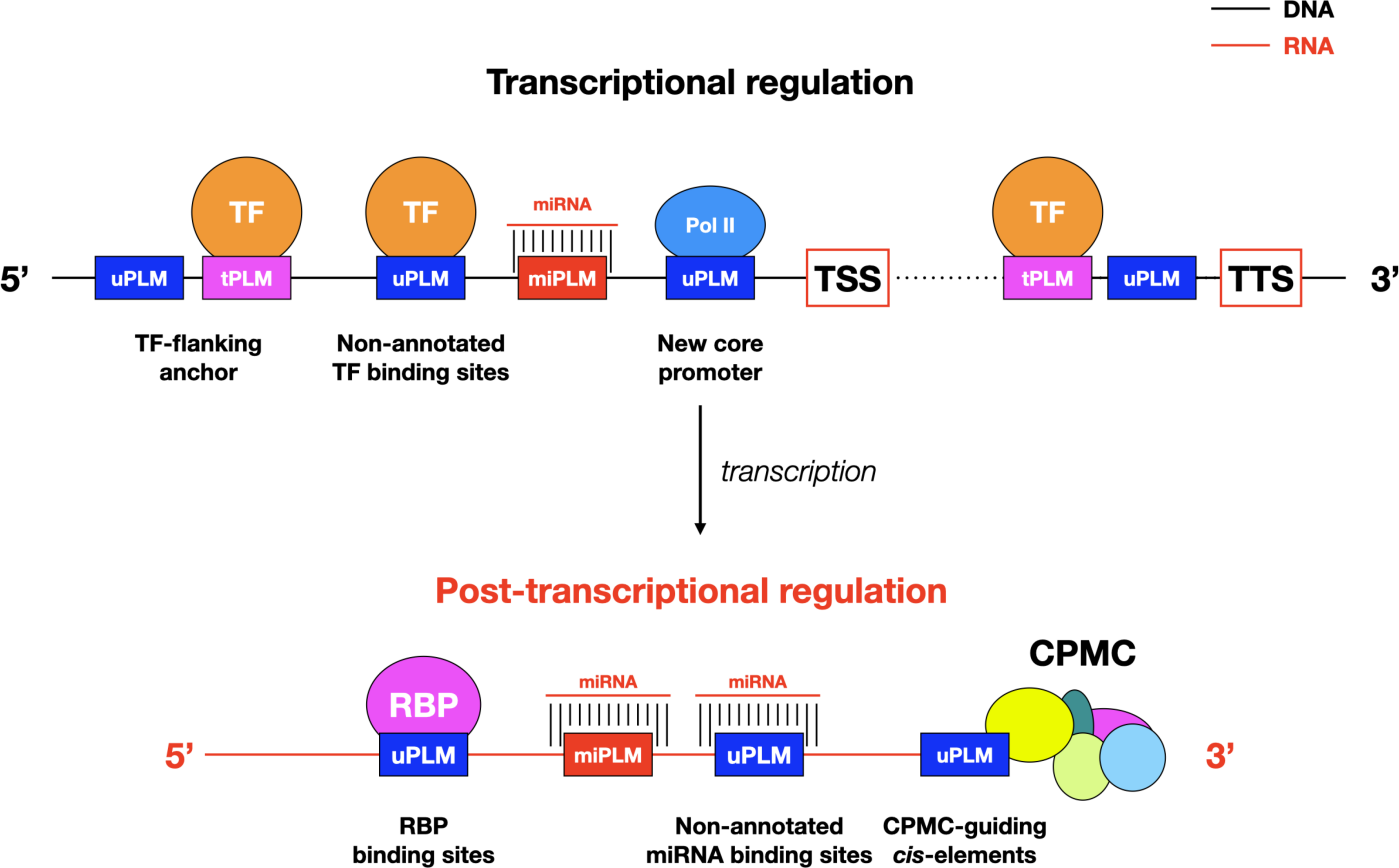
Models of PLM-mediated transcriptional and post-translational controls in plants. The models integrate only the results presented here. Possible interactions between the illustrated components and the diverse array of other intermediates such as enhancers and long-distance chromatin interaction sites, remain to be investigated. CPMC, cleavage and polyadenylation molecular complex; Pol II, RNA polymerase II; RPB: RNA binding proteins.

Understanding gene transcriptional regulation requires understanding where regulatory factors bind genomics DNA. Although several efforts have recently been undertaken to characterize TFBSs, the identification of high resolution *cis*-regulatory sequences at the genome-wide scale remains an arduous challenge. Hence, we attempted to reveal the whole PLM landscape by using genome-wide *de novo* PLM detection to systematically profile proximal putative *cis*-regulatory sequences. The three PLM group structure revealed in *A. thaliana* and *Z. mays* with distantly related genomes echoes and enriches established knowledge of the 5’-gene-proximal region (**Figure 8**). Omitting potential annotation errors, we found that the localization of these motifs, including the core promoter, was less constrained *in Z. mays* compared to *A. thaliana*, as recently reported for TATA-boxes at varying distances from TSS in *Z. mays* (Jores et al., 2021). Similarly, we observed that the dispersion of tPLMs remained more important in *Z. mays* than in *A. thaliana*, indicating that putative TFBSs have also a less constrained preferential localization in *Z. mays* than in *A. thaliana*. Overall, these data suggest that the 5’-proximal genomic context may be less constrained in *Z. mays* than in *A. thaliana*. This could be related to the richness of the *Z. mays* genome in transposable elements (TEs) compared with that of *A. thaliana* (Stitzer et al., 2021). TEs are known to be involved in regulating gene expression by introducing TFBSs into gene-proximal regions (Quesneville, 2020). Therefore, the study of TE-derived PLMs is an important perspective to obtain more information about PLMs and to better characterize the associated TEs, and deserves to be the subject of future studies.

Our finding of conserved PLMs between *A. thaliana* and *Z. mays* suggests that the closer we get to the genes, the more the context, including *cis*-regulatory elements (here given by tPLMs), are conserved between species. Notably, we have shown that this context appears to be more conserved in the 3’-gene-proximal region than in the 5’-gene proximal region: 14% of 3’-PLMs shared between the two species compared to 7% of 5’-PLMs (**Figure 2A**). This emphasize the importance of the 3’-gene-proximal region in genomic structure. Despite its key role in gene expression, the 3’-gene-proximal region remains poorly studied in plants (Srivastava et al., 2018; Mayr, 2019; Bernardes and Menossi, 2020). Because the density maxima observed for tPLMs and uPLMs was reached in the *cis*-elements that guide the CPMC and overlapped with groups 2 and 3, it is quite possible that the 3’-PLMs detected in these portions of DNA sequence constitute a catalog of NUE, FUE and CE (**Figure 8**). In support of this hypothesis, we observed that the AATAAA motif (and its complementary reverse), which is the key site involved in polyadenylation and is extremely conserved in mammals and somewhat less in plants, was located between 10 and 20 bases upstream of the TTS. Furthermore, the nucleotide percentages of 3’- PLMs detected in these regulatory regions are consistent with known proportions of nucleotides in these *cis*-elements. Together, these data support the idea that 3’-PLMs may constitute an accurate catalog of CPMC-guiding *cis*-elements. In this respect, the presence of 3’-tPLMs in this catalog (around 11%) and more generally in the whole region, opens interesting mechanistic perspectives on the role of TFs. First, they may act as activators or repressors of the transcriptional machinery (**Figure 8**). Second, by binding to tPLMs located in the FUE/NUE and CE regions, they could impact pre-mRNAs length and thus mRNA stability by influencing the choice of an alternative polyadenylation site at the end of transcripts (Srivastava et al., 2018; Mayr, 2019; Bernardes and Menossi, 2020).

The retained proportion observed between uPLMs (up to 25%) and tPLMs (up to 30%), also suggests that uPLMs could constitute a context that needs to be conserved. In this regard, the genomic significance of uPLMs is already supported by experimental maize ear MOA-seq data, suggesting that some of the identified uPLMs are potential TF footprints. It will be important to validate these uPLMs by functional assays to determine what proportion of them are indeed proximal *cis*-regulatory players. In addition, our finding raises the question of what mechanisms underlie the presence of uPLMs. First, as mentioned earlier, some uPLMs may be players in the core promoter or polyadenylation process (**Figure 8**). Second, some uPLMs may be non-annotated binding sites. Indeed, we showed that the 104 maize TF ChIP-seq data (Tu et al., 2020) contributed 16% and 33% more tPLMs for *A. thaliana* and *Z. mays*, respectively, compared to the JASPAR Plant 2020 database that was updated prior to the release of these ChIP-seq data. Similar to the post-transcriptional regulation by miRNAs, RNA-binding proteins (Lee and Kang, 2016; Cho et al., 2019) are major players that can potentially bind PLMs at the transcriptional level. Consequently, there is no doubt that future resources will supplement the assignment of uPLMs. Finally, uPLMs may be motifs that are not directly bound by TFs but that play a crucial role in the correct binding of these regulators to neighboring TFBSs (**Figure 8**) (Stringham et al., 2013; Crocker et al., 2015; Stampfel et al., 2015). This concept of “flanking sequence context” appears extremely relevant because of the nature of PLMs, which are constrained motifs at a distance from genes. This idea also raises many questions about the existence and role of tPLMs/uPLMs associations, with tPLMs bound by TFs and uPLMs serving as essential context sequences for the formation of the DNA-TF complex. Additional analyses and integration with other *in vivo* information will be key to advance functional tests needed to ascertain the relative importance of tPLMs and uPLMs as *cis*-regulatory elements controlling gene expression. Meanwhile, our results have broader implications for future characterization of unannotated genes in plants.

In summary, the implementation of the genome-wide and *de novo* PLMdetect method has demonstrated the richness of the gene-proximal regions and the interest in their further characterization. In particular, this work has highlighted the importance of the 3’-gene-proximal region as a major source of new knowledge and great interest for future studies.

## Methods

### Genomic datasets

TAIR10 (Lamesch et al., 2012) and B73v4.39 (Jiao et al., 2017) genomes and their annotations were considered to extract the 5’- and 3’-gene-proximal sequences of *A. thaliana* and *Z. mays* genes, respectively.

### Preparation of the gene-proximal sequence files

For the 5’-gene-proximal region, annotation of the TSS was ensured by filtering genes without a 5’-UTR region (in GFF3/GTF file). Genes on reverse strand were reverse-complemented to analyze all sequences in the same orientation. Extracted sequences corresponded to the intervals [-1000;+500] and [-1500;+500] bp relative to the TSS for *A. thaliana* and *Z. mays*, respectively. In total, 19,736 and 25,848 genes were analyzed for *A. thaliana* and *Z. mays*, respectively. For the 3’-gene-proximal region, similarly to what has been done for the 5’-gene-proximal region sequences, annotation of the TTS was ensured by filtering genes without a 3’-UTR region annotated. To standardize the PLM detection step, genes on forward strand were reverse-complemented. Extracted sequences were [-500;+1000] and [-500;+1500] bp with respect to the TTS for *A. thaliana* and *Z. mays*, respectively. Taking in consideration only annotated 3’-UTR, 20,573 and 25,199 genes were processed for *A. thaliana* and *Z. mays*, respectively.

### Preparation of the motif file

Every non-polymorphic DNA 4-mers to 8-mers was generated representing 87,296 motifs. Among these motifs, 256, 1,024, 4,096, 16,384, and 65,536 had a length of 4, 5, 6, 7 and 8 bp, respectively.

### Processing of potential PLM redundancy

To check PLM redundancy, we calculated the Jaccard index of each pair of PLMs for each PLM containing-gene set with an inclusion link. This index was obtained by dividing the intersection of the two lists by their union. A Jaccard index of 0.9 indicates an almost perfect match between the gene lists. This index was also calculated on the functional window of each pair of PLMs to quantify their overlap. We set the threshold for the functional window Jaccard index at 0.5.

### TFBS and microRNA resources and assignment

TFBSs (676 total) were extracted from JASPAR Plant 2020 (Fornes et al., 2019) and ChIP-seq of 104 maize leaf TFs (Tu et al., 2020). MicroRNAs (miRNAs) were obtained from psRNATarget (Dai et al., 2018) and only those from *A. thaliana* (427 total) and *Z. mays* (321 total) were kept.

We first assigned TFBSs to PLMs for both species in each region separately using the TOMTOM web tool (Gupta et al., 2007). Euclidean distance was next used as a comparison function with a q-value threshold at 0.05 and the complete scoring option deselected. PLMs were also compared to the top 1% of k-mers of the 104 maize TFs (Tu et al., 2020) by considering only exact matches. Because miRNAs regulate genes by sequence complementarity (Bartel, 2009) and are molecules from 19 to 22 nt, we only considered our 8 bp PLMs for this comparison (the 20 bp size by which miRNAs regulate gene expression was not tested due to computational time constraints). If a PLM was exactly found in a miRNA, it was assigned as a miPLM.

### Functional annotation

Functional annotation of genes from both species was based on MapMan X4 (Thimm et al., 2004) and Gene Ontology (GO) from PLAZA 4.5 (Van Bel et al., 2018). Functional enrichment analysis of genes containing an identified PLM (**Supplementary Datasets**) was performed by comparing the relative occurrence of each term to its relative occurrence in a reference list for each region and species using a hypergeometric test with the R function phyper. These reference lists consisted of all genes considered for PLM detection in each gene-proximal region in both species as described in the ‘Preparation of the gene-proximal sequence files’ of the Methods section. *P*-values were adjusted by the Benjamini-Hochberg (BH) procedure to control the False Discovery Rate (FDR). An enriched term had its adjusted *P*-value lower than 0.05.

### Comparative analysis of PLMs and MOA-seq motifs

*Z. mays* 5’-PLMs were compared to published MOA-seq motifs (Savadel et al., 2021) using TOMTOM with the same parameters as described for TFBSs assignment and according to the following two criteria: (1) both sequence types had to be identical (TOMTOM q-value <0.05) and (2) the position of the MOA-seq motif had to be within the functional window of the PLM extended by 30 bases upstream and downstream.

### Inference and topology analysis of the HOM04M002476 gene family regulatory network

A matrix of dimension 37x64 was generated with TF families in rows and HOM04M002476 genes in columns. Links between TF families and target genes were established when tPLMs, and thus associated TFs, were identified for a given target gene. These links were indicated by ones in the matrix. A zero indicated the absence of a tPLM associated with the TF family in the target gene. Gene and TF modules were obtained using LBM with the R package blockmodels (Leger, 2016).

Functional enrichment of each TF module was performed by comparing the relative occurrence of each term to its relative occurrence in the list of genes encoding TFs in *A. thaliana* (2,208 genes) and *Z. mays* (2,164 genes) using a hypergeometric test with the R phyper function. *P*-values were adjusted by the BH procedure to control the FDR. An enriched term had its adjusted *P*-value lower than 0.05.

## Data availability statement

The original contributions presented in the study are included in the article/**Supplementary Materials**. Further inquiries can be directed to the corresponding authors. Code used to generate the data are available at https://forgemia.inra.fr/GNet/plmdetect/plmdetect_tool.

## Author contributions

VB, M-LM and SC conceived the project. M-LM and SC designed and supervised the study. JR generated, analyzed and interpreted the data. CG formatted the functional annotation files. JR, M-LM and SC wrote the manuscript. All authors contributed to the article and approved the submitted version.

## Funding

This work was supported by the Plant2Pro^®^ Carnot Institute in the frame of the PLMViewer program. Plant2Pro^®^ is supported by ANR (agreement #19 CARN 0024 01). The IPS2 and IJPB laboratories benefit from the support of Saclay Plant Sciences-SPS (ANR-17-EUR-0007).

## Acknowledgments

We thank all members of the ‘Genomic Networks’ (GNet) and ‘Biomass Quality and Interactions with Drought’ (QUALIBIOSEC) teams past and present. We also thank Hank W. Bass for sharing some works prior to publication, Hervé Vaucheret for very helpful discussions, and two reviewers for comments that improved the manuscript.

## Conflict of interest

The authors declare that the research was conducted in the absence of any commercial or financial relationships that could be construed as a potential conflict of interest.

## Publisher’s note

All claims expressed in this article are solely those of the authors and do not necessarily represent those of their affiliated organizations, or those of the publisher, the editors and the reviewers. Any product that may be evaluated in this article, or claim that may be made by its manufacturer, is not guaranteed or endorsed by the publisher.

## References

[B1] Alonge M. Wang X. Benoit M. Soyk S. Pereira L. Zhang L.. (2020). Major impacts of widespread structural variation on gene expression and crop improvement in tomato. Cell 182, 145–161.e23. doi: 10.1016/j.cell.2020.05.021 32553272 PMC7354227

[B2] Axtell M. J. (2013). Classification and comparison of small RNAs from plants. Annu. Rev. Plant Biol. 64, 137–159. doi: 10.1146/annurev-arplant-050312-120043 23330790

[B3] Azodi C. B. Lloyd J. P. Shiu S.-H. (2020). The cis-regulatory codes of response to combined heat and drought stress in arabidopsis thaliana. NAR Genomics Bioinf. 2:lqaa049. doi: 10.1093/nargab/lqaa049 PMC7671360 33575601

[B4] Bartel D. P. (2009). MicroRNA target recognition and regulatory functions. Cell 136, 215–233. doi: 10.1016/j.cell.2009.01.002 19167326 PMC3794896

[B5] Bernard V. Brunaud V. Lecharny A. (2010). TC-motifs at the TATA-box expected position in plant genes: a novel class of motifs involved in the transcription regulation. BMC Genomics 11, 166. doi: 10.1186/1471-2164-11-166 20222994 PMC2842252

[B6] Bernardes W. S. Menossi M. (2020). Plant 3’ regulatory regions from mRNA-encoding genes and their uses to modulate expression. Front. Plant Sci. 11, 1252. doi: 10.3389/fpls.2020.01252 32922424 PMC7457121

[B7] Bernard V. Lecharny A. Brunaud V. (2010). Improved detection of motifs with preferential location in promoters. Genome 53, 739–752. doi: 10.1139/G10-042 20924423

[B8] Bueso E. Muñoz-Bertomeu J. Campos F. Brunaud V. Martínez L. Sayas E. (2014). ARABIDOPSIS THALIANA HOMEOBOX25 uncovers a role for gibberellins in seed Longevity1[C][W]. Plant Physiol. 164, 999–1010. doi: 10.1104/pp.113.232223 24335333 PMC3912122

[B9] Cho H. Cho H. S. Hwang I. (2019). Emerging roles of RNA-binding proteins in plant development. Curr. Opin. Plant Biol. 51, 51–57. doi: 10.1016/j.pbi.2019.03.016 31071564

[B10] Clément Y. Fustier M.-A. Nabholz B. Glémin S. (2014). The bimodal distribution of genic GC content is ancestral to monocot species. Genome Biol. Evol. 7, 336–348. doi: 10.1093/gbe/evu278 25527839 PMC4316631

[B11] Crocker J. Abe N. Rinaldi L. McGregor A. P. Frankel N. Wang S. (2015). Low affinity binding site clusters confer hox specificity and regulatory robustness. Cell 160, 191–203. doi: 10.1016/j.cell.2014.11.041 25557079 PMC4449256

[B12] Dai X. Zhuang Z. Zhao P. X. (2018). psRNATarget: a plant small RNA target analysis server (2017 release). Nucleic Acids Res. 46, W49–W54. doi: 10.1093/nar/gky316 29718424 PMC6030838

[B13] Fagny M. Kuijjer M. L. Stam M. Joets J. Turc O. Rozière J.. (2021). Identification of key tissue-specific, biological processes by integrating enhancer information in maize gene regulatory networks. Front. Genet. 11, 606285. doi: 10.3389/fgene.2020.606285 33505431 PMC7834273

[B14] Fornes O. Castro-Mondragon J. A. Khan A. van der Lee R. Zhang X. Richmond P. A.. (2019). JASPAR 2020: update of the open-access database of transcription factor binding profiles. Nucleic Acids Res. 48, D87–D92. doi: 10.1093/nar/gkz1001 PMC7145627 31701148

[B15] Frei dit Frey N.. (2014). Functional analysis of arabidopsis immune-related MAPKs uncovers a role for MPK3 as negative regulator of inducible defences. Genome Biol. 15, R87. doi: 10.1186/gb-2014-15-6-r87 24980080 PMC4197828

[B16] Grosschedl R. Birnstiel M. L. (1980). Identification of regulatory sequences in the prelude sequences of an H2A histone gene by the study of specific deletion mutants *in vivo* . PNAS 77, 1432–1436. doi: 10.1073/pnas.77.3.1432 6929494 PMC348509

[B17] Gupta S. Stamatoyannopoulos J. A. Bailey T. L. Noble W. S. (2007). Quantifying similarity between motifs. Genome Biol. 8, R24. doi: 10.1186/gb-2007-8-2-r24 17324271 PMC1852410

[B18] Hammal F. de Langen P. Bergon A. Lopez F. Ballester B. (2022). ReMap 2022: a database of human, mouse, drosophila and arabidopsis regulatory regions from an integrative analysis of DNA-binding sequencing experiments. Nucleic Acids Res. 50, D316–D325. doi: 10.1093/nar/gkab996 34751401 PMC8728178

[B19] Jiao Y.. (2017). Improved maize reference genome with single-molecule technologies. Nature 546, 524–527. doi: 10.1038/nature22971 28605751 PMC7052699

[B20] Jores T.. (2021). Synthetic promoter designs enabled by a comprehensive analysis of plant core promoters. Nat. Plants 7, 842–855. doi: 10.1038/s41477-021-00932-y 34083762 PMC10246763

[B21] Joshi C. P. (1987). An inspection of the domain between putative TATA box and translation start site in 79 plant genes. Nucleic Acids Res. 15, 6643–6653. doi: 10.1093/nar/15.16.6643 3628002 PMC306128

[B22] Ksouri N. Castro-Mondragón J. A. Montardit-Tarda F. van Helden J. Contreras-Moreira B. Gogorcena Y. (2021). Tuning promoter boundaries improves regulatory motif discovery in nonmodel plants: the peach example. Plant Physiol. 185, 1242–1258. doi: 10.1093/plphys/kiaa091 33744946 PMC8133646

[B23] Lai X. Stigliani A. Vachon G. Carles C. Smaczniak C. Zubieta C.. (2019). Building transcription factor binding site models to understand gene regulation in plants. Mol. Plant 12, 743–763. doi: 10.1016/j.molp.2018.10.010 30447332

[B24] Lamesch P. Berardini T. Z. Li D. Swarbreck D. Wilks C. Sasidharan R.. (2012). The arabidopsis information resource (TAIR): improved gene annotation and new tools. Nucleic Acids Res. 40, D1202–D1210. doi: 10.1093/nar/gkr1090 22140109 PMC3245047

[B25] Lee K. Kang H. (2016). Emerging roles of RNA-binding proteins in plant growth, development, and stress responses. Mol. Cells 39, 179–185. doi: 10.14348/molcells.2016.2359 26831454 PMC4794599

[B26] Leger J.-B. (2016). Blockmodels: A r-package for estimating in latent block model anpd stochastic block model, with various probability functions, with or without covariates. arXiv:1602.07587 [stat]. doi: 10.48550/arXiv.1602.07587

[B27] Li X. Zhu C. Yeh C.-T. Wu W. Takacs E. M. Petsch K. A.. (2012). Genic and nongenic contributions to natural variation of quantitative traits in maize. Genome Res. 22, 2436–2444. doi: 10.1101/gr.140277.112 22701078 PMC3514673

[B28] Liu S. Li C. Wang H. Wang S. Yang S. Liu X.. (2020). Mapping regulatory variants controlling gene expression in drought response and tolerance in maize. Genome Biol. 21, 163. doi: 10.1186/s13059-020-02069-1 32631406 PMC7336464

[B29] Martínez F. Arif A. Nebauer S. G. Bueso E. Ali R. Montesinos C.. (2015). A fungal transcription factor gene is expressed in plants from its own promoter and improves drought tolerance. Planta 242, 39–52. doi: 10.1007/s00425-015-2285-5 25809153

[B30] Mayr C. (2019). What are 3′ UTRs doing? Cold Spring Harb. Perspect. Biol. 11, a034728. doi: 10.1101/cshperspect.a034728 30181377 PMC6771366

[B31] Molina C. Grotewold E. (2005). Genome wide analysis of arabidopsis core promoters. BMC Genomics 6, 25. doi: 10.1186/1471-2164-6-25 15733318 PMC554773

[B32] Ossowski S. Schwab R. Weigel D. (2008). Gene silencing in plants using artificial microRNAs and other small RNAs: Engineering small RNA-mediated gene silencing. Plant J. 53, 674–690. doi: 10.1111/j.1365-313X.2007.03328.x 18269576

[B33] Peng F. Y. Hu Z. Yang R.-C. (2016). Bioinformatic prediction of transcription factor binding sites at promoter regions of genes for photoperiod and vernalization responses in model and temperate cereal plants. BMC Genomics 17, 573. doi: 10.1186/s12864-016-2916-7 27503086 PMC4977670

[B34] Quesneville H. (2020). Twenty years of transposable element analysis in the arabidopsis thaliana genome. Mobile DNA 11, 28. doi: 10.1186/s13100-020-00223-x 32742313 PMC7385966

[B35] Savadel S. D. Hartwig T. Turpin Z. M. Vera D. L. Lung P.-Y. Sui X.. (2021). The native cistrome and sequence motif families of the maize ear. PloS Genet. 17, e1009689. doi: 10.1371/journal.pgen.1009689 34383745 PMC8360572

[B36] Schmitz R. J. Grotewold E. Stam M. (2021). Cis-regulatory sequences in plants: Their importance, discovery, and future challenges. Plant Cell 34, 718–741. doi: 10.1093/plcell/koab281 PMC8824567 34918159

[B37] Srivastava A. K. Lu Y. Zinta G. Lang Z. Zhu J.-K. (2018). UTR dependent control of gene expression in plants. Trends Plant Sci. 23, 248–259. doi: 10.1016/j.tplants.2017.11.003 29223924 PMC5828884

[B38] Stampfel G. Kazmar T. Frank O. Wienerroither S. Reiter F. Stark A. (2015). Transcriptional regulators form diverse groups with context-dependent regulatory functions. Nature 528, 147–151. doi: 10.1038/nature15545 26550828

[B39] Stitzer M. C. Anderson S. N. Springer N. M. Ross-Ibarra J. (2021). The genomic ecosystem of transposable elements in maize. PloS Genet. 17, e1009768. doi: 10.1371/journal.pgen.1009768 34648488 PMC8547701

[B40] Stringham J. L. Brown A. S. Drewell R. A. Dresch J. M. (2013). Flanking sequence context-dependent transcription factor binding in early drosophila development. BMC Bioinf. 14, 298. doi: 10.1186/1471-2105-14-298 PMC3851692 24093548

[B41] Sundararajan A. Dukowic-Schulze S. Kwicklis M. Engstrom K. Garcia N. Oviedo O. J.. (2016). Gene evolutionary trajectories and GC patterns driven by recombination in zea mays. Front. Plant Sci. 7, 1433. doi: 10.3389/fpls.2016.01433 27713757 PMC5031598

[B42] Tarutani Y. Shiba H. Iwano M. Kakizaki T. Suzuki G. Watanabe M.. (2010). Trans-acting small RNA determines dominance relationships in brassica self-incompatibility. Nature 466, 983–986. doi: 10.1038/nature09308 20725042

[B43] Thimm O. Bläsing O. Gibon Y. Nagel A. Meyer S. Krüger P.. (2004). MAPMAN: a user-driven tool to display genomics data sets onto diagrams of metabolic pathways and other biological processes. Plant J. 37, 914–939. doi: 10.1111/j.1365-313X.2004.02016.x 14996223

[B44] Tu X. Mejía-Guerra M. K. Franco J. A.V. Tzeng D. Chu P.-Y. Shen W.. (2020). Reconstructing the maize leaf regulatory network using ChIP-seq data of 104 transcription factors. Nat. Commun. 11, 1–13. doi: 10.1038/s41467-020-18832-8 33037196 PMC7547689

[B45] Van Bel M. Diels T. Vancaester E. Kreft L. Botzki A. Van de Peer Y.. (2018). PLAZA 4.0: an integrative resource for functional, evolutionary and comparative plant genomics. Nucleic Acids Res. 46, D1190–D1196. doi: 10.1093/nar/gkx1002 29069403 PMC5753339

[B46] Wallace J. G. Bradbury P. J. Zhang N. Gibon Y. Stitt M. Buckler E. S. (2014). Association mapping across numerous traits reveals patterns of functional variation in maize. PloS Genet. 10, e1004845. doi: 10.1371/journal.pgen.1004845 25474422 PMC4256217

[B47] Wang Y. Fairley J. A. Roberts S. G. E. (2010). Phosphorylation of TFIIB links transcription initiation and termination. Curr. Biol. 20, 548–553. doi: 10.1016/j.cub.2010.01.052 20226668 PMC2849011

[B48] Waters A. J. Makarevitch I. Noshay J. Burghardt L. T. Hirsch C. N. Hirsch C. D.. (2017). Natural variation for gene expression responses to abiotic stress in maize. Plant J. 89, 706–717. doi: 10.1111/tpj.13414 28188666

[B49] Yamamoto Y. Y. Ichida H. Abe T. Suzuki Y. Sugano S. Obokata J. (2007). Differentiation of core promoter architecture between plants and mammals revealed by LDSS analysis. Nucleic Acids Res. 35, 6219–6226. doi: 10.1093/nar/gkm685 17855401 PMC2094075

[B50] Yu C.-P. Lin J.-J. Li W.-H. (2016). Positional distribution of transcription factor binding sites in arabidopsis thaliana. Sci. Rep. 6, 25164. doi: 10.1038/srep25164 27117388 PMC4846880

[B51] Zemlyanskaya E. V. Dolgikh V. A. Levitsky V. G. Mironova V. (2021). Transcriptional regulation in plants: Using omics data to crack the cis-regulatory code. Curr. Opin. Plant Biol. 63, 102058. doi: 10.1016/j.pbi.2021.102058 34098218

[B52] Zhou P. Enders T. A. Myers Z. A. Magnusson E. Crisp P. A. Noshay J. M.. (2022). Prediction of conserved and variable heat and cold stress response in maize using cis-regulatory information. Plant Cell 34, 514–534. doi: 10.1093/plcell/koab267 34735005 PMC8773969

