# Supplementary material for: A comprehensive map of preferentially located motifs reveals distinct proximal cis-regulatory sequences in plants
Source: Front Plant Sci. 2022 Oct 12;13:976371. doi: 10.3389/fpls.2022.976371 (PMC9597372; doi:10.3389/fpls.2022.976371)
Supplement: Supplementary file 2 [file DataSheet_2.pdf]

The Supplementary Datasets are accessible via this link:
https://forgemia.inra.fr/GNet/plmdetect/plmdetect_tool/-/blob/main/Supplementary%20Data_Roziere_et_al.zip
